# Photodynamic Therapy of Ovarian Carcinoma Cells with Curcumin-Loaded Biodegradable Polymeric Nanoparticles

**DOI:** 10.3390/pharmaceutics11060282

**Published:** 2019-06-15

**Authors:** Lili Duse, Michael Rene Agel, Shashank Reddy Pinnapireddy, Jens Schäfer, Mohammed A. Selo, Carsten Ehrhardt, Udo Bakowsky

**Affiliations:** 1 Department of Pharmaceutics and Biopharmaceutics, University of Marburg, Robert-Koch-Str. 4, 35037 Marburg, Germany; lili.duse@pharmazie.uni-marburg.de (L.D.); michael.agel@pharmazie.uni-marburg.de (M.R.A.); shashank.pinnapireddy@pharmazie.uni-marburg.de (S.R.P.); j.schaefer@staff.uni-marburg.de (J.S.); 2 School of Pharmacy and Pharmaceutical Sciences and Biomedical Sciences Institute, Trinity College Dublin, Dublin 2, Ireland; mohammeda.mohsin@uokufa.edu.iq (M.A.S.); ehrhardc@tcd.ie (C.E.); 3 Faculty of Pharmacy, University of Kufa, 31001 Kufa, Iraq

**Keywords:** PLGA, nanoscaled drug delivery, LED, cancer, serum stability, reactive oxygen species, cellular uptake

## Abstract

Accumulation of photosensitisers in photodynamic therapy in healthy tissues is often the cause of unwanted side effects. Using nanoparticles, improved bioavailability and site-specific drug uptake can be achieved. In this study, curcumin, a natural product with anticancer properties, albeit with poor aqueous solubility, was encapsulated in biodegradable polymeric poly(lactic-*co*-glycolic acid) (PLGA) nanoparticles (CUR-NP). Dynamic light scattering, laser Doppler anemometry and atomic force microscopy were used to characterise the formulations. Using haemolysis, serum stability and activated partial thromboplastin time tests, the biocompatibility of CUR-NP was assessed. Particle uptake and accumulation were determined by confocal laser scanning microscopy. Therapeutic efficacy of the formulation was tested in SK-OV-3 human ovarian adenocarcinoma cells post low level LED irradiation by determining the generation of reactive oxygen species and cytotoxicity. Pharmacologic inhibitors of cellular uptake pathways were used to identify the particle uptake mechanism. CUR-NP exhibited better physicochemical properties such as stability in the presence of light and improved serum stability compared to free curcumin. In addition, the novel nanoformulation facilitated the use of higher amounts of curcumin and showed strong apoptotic effects on tumour cells.

## 1. Introduction

Light has long been used in the treatment of diseases as different as psoriasis, rickets or cancer [1]. With an alarming mortality rate, cancer is one of the most prevalent causes of death worldwide [2]. Hence, tremendous efforts have been made to develop novel safe, selective and effective cancer therapies. One such treatment, photodynamic therapy, exemplifies a novel minimally invasive tumour targeting therapy, in which tumour tissues can be selectively destroyed by three main mechanisms [3,4]. In the first case there is a combination of three individually inert entities viz. a photoactive drug molecule (photosensitiser; PS), oxygen and a light source of a specific wavelength, which upon combining, activate the photosensitiser and exhibit toxicity towards cancer cells and tissues [5,6]. This process of photosensitisation hugely relies upon the presence of molecular oxygen and forms the basis for a successful photodynamic therapy (PDT) [7]. After absorbing energy from the light source, the PS interacts with molecular oxygen via energy transfer process or electron transfer process and generates reactive oxygen species (ROS), which oxidise cellular and subcellular organelles to induce either apoptosis or necrosis leading to cell death [8,9]. Since cytotoxic ROS occurs only after irradiation of the PS, tumour tissues can be targeted with high precision. Due to its high reactivity and short half-life, ROS generation only targets cells lying in close proximity of the site of irradiation [1]. The half-life of singlet oxygen in biological systems is ~0.01–0.04 µs, with a restricted site of action of a radius spanning 0.01–0.02 µm [5,10]. PDT can also damage the tumour-associated vasculature, leading to tumour infarction. Finally, PDT can activate the immune response against tumour cells. All three mentioned mechanisms can also influence each other and act synergistically. PDT has an added advantage of almost no or minimal effect towards healthy tissues, making this therapy site specific [11]. It could also be combined with other therapies or immune-stimulatory agents like microbial adjuvants or cytokine for T-cell therapy and adoptive cellular therapy, respectively [1]. Another advantage of PDT is its effectiveness against otherwise chemoresistant cell types [12]. Furthermore, the photosensitiser can be administered by various means, such as systemically, locally or topically [1].

For the success of any PDT, it is important to choose an optimal PS. Despite many studies performed using different PS, only a few have reached the stage of advanced human clinical trials or even U.S. Food and Drug Administration (FDA) approval for clinical use [13]. A very promising photosensitiser for photodynamic treatment of tumours is curcumin. Curcumin (diferuloylmethane), is a naturally occurring yellowish polyphenol extract from the rhizomes of turmeric (*Curcuma longa*), which is cultivated widely in south and southeast tropical Asia [14]. Curcumin is the most active component of turmeric, making up approx. 0.5–3.14% of the dry weight of turmeric powder. Curcumin is used for a variety of therapeutic activities against many different diseases and conditions such as skin diseases, pulmonary and gastrointestinal systems, aches, pains, wounds, sprains, and liver disorders [15].

Offering a variety of potential applications, curcumin has been commonly used as a food-colouring agent (E100) as well as in pharmaceutical research due to its anti-inflammatory, anti-oxidative, anti-carcinogenic and anti-microbial effects [16,17]. It is a proven anticancer agent and therefore a perfect choice for a photosensitiser [18]. However, curcumin exhibits a very low level of bioavailability, since it is a highly lipophilic substance (solubility 11 ng/mL in phosphate buffer saline, PBS; pH 5) [19]. Systemically absorbed curcumin is prone to an extensive first-pass metabolism and undergoes a fast metabolic reduction with biliary excretion [20]. In 1978, Wahlstrom and Blennow observed the uptake of curcumin using Sprague–Dawley rats. After oral administration of 1 g/kg curcumin only negligible amounts of curcumin could be found in blood plasma [21].

These limitations can be overcome by the use of polymeric nanoparticles such as carrier systems, wherein the active substance is protected from degradation in the physiological environment and the bio-membrane permeability and cellular uptake of highly hydrophobic molecules are facilitated [17]. Several studies have revealed a remarkable enhancement of curcumin’s phototoxicity through nanoparticle encapsulation [22,23,24,25,26]. One such polymer is PLGA, which is an FDA and European Medicines Agency (EMA) approved biocompatible and biodegradable polymer [27,28]. In the body, PLGA undergoes hydrolysis wherein the endogenous metabolite monomers lactic acid and glycolic acid are and easily metabolised by the body (to water and carbon dioxide) via Krebs cycle [27,29,30]. The degradation time of PLGA and the release of drug from the nanoparticles mainly depends on the nature of copolymer composition [31]. In this study, PLGA 50:50 was chosen due to its ability to hydrolyse much faster than other types of PLGA with a half-life (50% loss of molecular weight) of 15 days for microspheres [32,33]. 

Another important aspect in PDT is the choice of the irradiation device. There is an array of radiation devices available for PDT such as lasers or lamps [13]. In the current study, with an aim to make PDT more cost effective, efficient and safe, we have utilised a custom manufactured prototype low level light emitting diode (LED) array for the irradiation of PS [34]. LEDs are energy efficient, generate less thermal energy in the form of heat and are versatile in terms of structural form. They have the ability to emit light over a wide area with a relatively homogenous output, which enables treatment of larger lesions in fewer therapy sessions. Using a combination of curcumin loaded PLGA nanoparticles and LED-based PDT, superficial tumours (melanomas and lymphomas) and accessible adenocarcinomas (such as ovarian and cervical) could be treated effectively. The PLGA nanoparticles can be administered intratumourally or could be surface modified to enable systemic application.

In the present study, we have exploited the combination of biodegradable PLGA nanoparticles and LED-based PDT using curcumin as a photosensitiser. The nanoparticles have been characterised for their size and surface charge using Dynamic light scattering (DLS) and laser Doppler anemometry (LDA), respectively. Structural morphology was analysed using atomic force microscopy (AFM) and transmission electron microscopy (TEM). The photo-destructive effects have been evaluated in vitro by cytotoxicity assays and confocal laser scanning microscopy (CLSM). The cellular uptake in SK-OV-3 cells was also analysed using three different uptake inhibitors. Irradiation experiments were carried out in SK-OV-3 tumour cells and the oxidative stress induced during the irradiation was analysed by determining the inhibition of ROS. Furthermore, haemolysis assays using fresh blood, serum stability using serum and activated partial thromboplastin time tests (aPTT) using plasma were used to demonstrate the biocompatibility of the curcumin-loaded polymeric nanoparticles.

## 2. Materials and Methods

### 2.1. Materials

Curcumin (95% purity) was obtained from Alfa Aesar (Ward Hill, MA, USA); PLGA (Resomer RG 503 H) was supplied by Evonik (Essen, Germany); poly(vinyl alcohol) (PVA, Mowiol 4-88) was purchased from Kuraray (Hattersheim, Germany); polysorbate 80 (Tween 80), 3-(4,5-dimethylthiazol-2-yl)-2,5-diphenyltetrazolium bromide (MTT), 2’,7’-dichlorofluorescin diacetate (DCFDA) and tert-butyl hydroperoxide (TBHP) were obtained from Sigma-Aldrich Chemie (Taufkirchen, Germany). Inhibitors of cellular uptake, viz. Filipin III, chlorpromazine and dynasore were also obtained from Sigma Aldrich. Ultrapure water, generated by a PURELAB flex 4 device (ELGA LabWater, High Wycombe, UK) was used for all experiments. For cell culture studies, ultrapure water was additionally autoclaved and filter-sterilised using 0.2 μm polyethersulphone membrane filters (Sarstedt, Nümbrecht, Germany) prior to use. HPLC-grade ethyl acetate (VWR, Darmstadt, Germany) was used to prepare the organic phases. All other chemicals used were of analytical grade. All buffers used in this study were prepared in the laboratory, unless stated otherwise.

### 2.2. Light Source

The LED device used in this study was custom made by Lumundus (Eisenach, Germany) to be usable with microtiter plates. The device is equipped with an array of two different LEDs, capable of emitting light at wavelengths of 457 nm and 620 nm, respectively. Irradiation time, current (i.e., 20, 40, 60, 80, and 100 mA) and wavelength settings are adjustable as per the energy requirement. Radiation intensity was calculated based on the current and irradiation time. 

### 2.3. Cell culture

The human ovarian adenocarcinoma cell line SK-OV-3 was procured from American Type Culture Collection (ATCC, Manassas, VA, USA). SK-OV-3 cells were cultivated in Iscove’s modified Dulbecco’s medium (Capricorn Scientific, Ebsdorfergrund, Germany) supplemented with 10% foetal bovine serum (Sigma-Aldrich, Taufkirchen, Germany) at 37 °C and 7% CO_2_ in humidified atmosphere. The medium was replaced every other day and the cells were sub-cultured upon reaching 80% confluency.

### 2.4. Formulation of Nanoparticles

Curcumin-loaded nanoparticles (CUR-NP) and unloaded PLGA nanoparticles (NP) were prepared from PLGA by the emulsion–diffusion–evaporation technique as previously described by Kumar et al. [35]. In a pilot study, the method was optimised by varying formulation parameters (e.g., concentration of PLGA, PVA and curcumin; homogenisation speed and time). Briefly, 200 mg of PLGA were dissolved in 5 mL ethyl acetate at room temperature. Curcumin stock solution was prepared by dissolving curcumin in ethyl acetate (2 mg/mL). Equal volumes of PLGA and curcumin stock solutions were mixed and filtered through a 0.45 µm nylon syringe filter (Pall Corporation, New York, NY, USA). The organic solution was added dropwise to an aqueous solution of 2% PVA (*w*/*w*) and the resulting emulsion was stirred in a sealed tube for about 3 h, before homogenising for 10 min at 13,400 rpm using an Ultra-Turrax T25 homogeniser (IKA-Werke, Staufen, Germany). Nanoprecipitation was induced by adding water at a constant rate of 120 mL/h using a syringe pump (Perfusor, B. Braun, Melsungen, Germany). Finally, the organic solvent was evaporated by stirring overnight at room temperature in a dark environment. The nanosuspension was adjusted to a final volume of 50 mL and a concentration of 0.1 mg/mL curcumin. Unloaded nanoparticles were prepared by the same protocol, except that pure ethyl acetate was used instead of the curcumin stock solution. To remove agglomerates, the nanoformulations were centrifuged at 2000× *g* for 45 s using an Eppendorf Centrifuge 5418 (Eppendorf, Hamburg, Germany). The resulting pellet was discarded, and the supernatant was washed three times with water to separate the nanospheres from non-encapsulated curcumin. Between each washing step, centrifugation at 16,000× *g* for 45 min was performed. For prolonged storage, the CUR-NP were freeze-dried using an Alpha 1-4LSC lyophiliser (Martin Christ Gefriertrocknungsanlagen, Osterode am Harz, Germany) using 0.5% PVA as a cryoprotectant. Afterwards the nanoformulations were stored at 4 °C protected from light.

### 2.5. Dynamic Light Scattering and Laser Doppler Anemometry

To determine particle size distribution and zeta (ζ)-potential of the particles, DLS and LDA were used, respectively (Zetasizer Nano ZS, Malvern Instruments, Malvern, UK). A viscosity of 0.88 mPa × s and a refractive index of 1.33 of water at 25 °C were assumed for data interpretation. Measurement position and laser attenuation were automatically adjusted by the instrument. The instrument performs 15 size runs per measurement with each lasting 10 s. For ζ-potential measurements, the instrument automatically performs 15–100 runs per measurement, depending upon the sample. All samples were diluted with filtered PBS buffer (1:100) [36]. Data from at least three independent experiments were measured for both DLS and LDA analysis.

### 2.6. Morphological Characterisation

To investigate the morphology of CUR-NP and to confirm size data obtained by DLS measurements, AFM was performed using a Nanowizard 3® (JPK Instruments, Berlin, Germany) and a Digital Nanoscope IV Bioscope (Veeco Instruments, Santa Barbara, CA, USA), respectively. Formulations were diluted 1:100 with water and 20 µL of the diluted sample were placed onto a silica wafer or an untreated microscopic glass slide. The samples were left to settle onto the surface for a few minutes and the remaining fluid was absorbed by a lint-free wipe (Kimtech Precision Wipes, Kimberly-Clark, Fullerton, CA, USA). Measurements were performed in tapping mode, in which the cantilever oscillated with determined amplitude close to its resonance frequency, with scan rates from 0.5 to 1 Hz. A HQ:NSC16/AL_BS (Anfatec Instruments, Oelsnitz, Germany) cantilever was used [37]. Data were processed using JPKSPM data processing software (v. 5.1.8, JPK instruments). 

For the TEM analysis, the nanoparticle suspension was applied onto 300-mesh copper grids. Samples were then negative stained thrice with 2% uranyl acetate (pH 4.2), which was alternated by washing steps with water. The samples were then allowed to dry overnight before being examined under the TEM (LEO 912 AB, Carl Zeiss, Jena, Germany) [22].

### 2.7. Yield, Encapsulation Efficiency and Loading Capacity

The freeze-dried nanoparticles were weighted, and the percentage yield was calculated using the following equation

(1) $$\%\mathrm{Yield}=\frac{\mathrm{Dry}\;\mathrm{weight}\;\mathrm{of}\;\mathrm{nanoparticles}}{\mathrm{Weight}\;\mathrm{of}\;\mathrm{drug}\;\mathrm{and}\;\mathrm{polymer}\;\mathrm{used}\;\mathrm{for}\;\mathrm{NP}\;\mathrm{preparation}}\times 100$$

The encapsulation efficiency (%EE) was determined by extracting curcumin from nanospheres that were previously washed and separated from free curcumin, as previously described in Section 2.4. The nanosuspension was mixed with an equal volume of acetonitrile (a solvent for PLGA and curcumin) and sonicated for 20 min. The absorbance of this solution was measured spectrophotometrically at 425 nm using a Multiskan GO micro plate reader (Thermo Fischer Scientific, Waltham, MA, USA), and the amount of drug quantified using a calibration curve recorded with known curcumin concentrations. For determination of the total drug content, nanoparticles without any washing procedure were measured in the same way. The %EE (*w*/*w*) was calculated as follows:

(2) $$\%\mathrm{EE}=\frac{{\mathrm{Drug}}_{\mathrm{encapsulated}}}{{\mathrm{Drug}}_{\mathrm{total}}}\times 100$$

In addition, the loading capacity (LC) was calculated using the equation:

(3) $$\%\mathrm{LC}=\frac{{\mathrm{Drug}}_{\mathrm{encapsulated}}}{\mathrm{Dry}\;\mathrm{weight}\;\mathrm{of}\;\mathrm{nanoparticles}}\times 100$$

### 2.8. In Vitro Drug Release

For determination of drug release, 10 mg lyophilised nanoparticles were redispersed in 30 mL PBS buffer (pH 5.5 and pH 7.4) containing 1% polysorbate 80 (*v*/*v*) to assure sink conditions. This dispersion was stored under absence of light in a shaking incubator (TH 15/KS 15, Edmund Bühler, Bodelshausen, Germany) at 37 °C under slight agitation (50 rpm). For the following seven days, 500 µL samples were drawn and replaced with fresh medium. The samples were centrifuged for 10 min at 16,000× *g* (Centrifuge 5418, Eppendorf AG) and the amount of curcumin present in the supernatant was determined spectrophotometrically at 425 nm, using Multiskan GO micro plate reader (Thermo Fischer Scientific).

### 2.9. Serum Stability

To study the stability of the formulations in physiologically relevant conditions, 2 mL serum was diluted with 20 mM HEPES (pH 7.4) to make a 60% serum solution. Curcumin-loaded PLGA nanoparticles were added in a ratio of 1:5 (*v*/*v*). The mixture was incubated at 37 °C in a shaking incubator at 100 rpm for 24 h. Samples were further diluted to a ratio of 1:20 (*v*/*v*) with 20 mM HEPES (pH 7.4) prior to DLS and LDA analyses at different time points. All measurements were carried out in triplicates [38].

### 2.10. In Vitro Irradiation Experiments

Cells were seeded onto in 96-well plates (10,000 cells/0.35 cm^2^ (per well); Nunclon Delta, Thermo Fisher Scientific, Waltham, MA, USA) and were allowed to adhere overnight. Various concentrations of CUR-NP and free curcumin (dissolved in DMSO) were added to the wells and incubated for 4 h. Irradiation was performed at a wavelength of 457 nm with a radiation fluence of 8.6 J/cm^2^ and the plates were incubated overnight. Dark (un-irradiated) plates were used as control [39]. Subsequently, the medium was replaced with fresh medium containing MTT dye and cells were incubated for 4 h. Absorbance of the resulting formazan crystals dissolved in DMSO was measured using a plate reader (FLUOstar Optima; BMG Labtech, Offenburg, Germany) at 570 nm. Viability of untreated cells was considered as 100%.

### 2.11. Cellular Uptake Studies

To determine the cellular uptake mechanism, SK-OV-3 cells were seeded at a density of 10,000 cells/0.35 cm^2^ (per well) in 96-well plates (Nunclon Delta, Nunc/Thermo Fischer Scientific, Waltham, MA, USA) and incubated overnight. The next day, cells were washed with PBS containing Ca^2+^ and Mg^2+^ (pH 7.4) and were incubated for 30 min with pharmacological inhibitors of different endocytic mechanisms viz. dynasore (80 µM), chlorpromazine (14 µM) and Filipin III (8 mM) [40]. After incubation with the uptake inhibitors, cells were washed again with PBS and exposed to 50 µM CUR-NP or free curcumin (dissolved in DMSO) for 4 h. After irradiation at 457 nm with a radiation fluence of 8.6 J/cm^2^, cells were incubated with MTT and the absorbance from resulting formazan crystals (dissolved in DMSO) was determined at 570 nm as described above.

### 2.12. Reactive Oxygen Species

ROS were determined using 2’,7’-dichlorofluorescin diacetate (DCFDA, Abcam, Cambridge, UK) according to the supplier’s protocol with slight modifications [34]. Briefly, SK-OV-3 cells were seeded in 96-well plates as mentioned above. The next day, cells were incubated with CUR-NP for 4 h. Tert-butyl hydroperoxide (TBHP, 50 µM) was used as positive control. Cells were subsequently washed using PBS containing Ca^2+^ and Mg^2+^ (pH 7.4) and supplemented with fresh medium. Irradiation was carried out at 457 nm with a radiation fluence of 8.6 J/cm^2^. The cells were washed again with PBS and incubated with culture medium (IMDM without phenol red) containing 25 µM DCFDA for 1 h. Cell culture lysis reagent (Promega, Mannheim, Germany) was used to lyse the cell and fluorescence was recorded using a FLUOstar Optima plate reader (λ_ex_ 480 nm/λ_em_ 520 nm).

### 2.13. Intracellular Visualisation of PDT

Cells were seeded onto 12-well plates (90,000 cells/3.5 cm^2^ (per well); Nunclon Delta) containing cover slips (15 mm in diameter). The plates were incubated for 24 h, before being used for the experiments. CUR-NP suspension was added dropwise to each well. After 4 h, the supernatant was removed and cells were washed twice with PBS containing Ca^2+^ and Mg^2+^ (pH 7.4). Cell were treated with a 4% formaldehyde solution for 20 min to fix them. The nucleus was counterstained with 0.1 µg/mL 4’,6-diamidino-2-phenylindole (DAPI) for 20 min. Finally, the cells were washed again with PBS (pH 7.4) and the cover slips were mounted onto slides. Samples were examined under a LSM700 confocal laser-scanning microscope (Carl Zeiss Microscopy, Jena, Germany). 

### 2.14. Activated Partial Thromboplastin Time Test

An aPTT test was performed as previously described, using a Coatron M1 coagulation analyser (Teco, Neufahrn, Germany) to determine the effect of the formulations on blood coagulation [41]. Briefly, 25 µL of plasma was mixed with 25 µL each of sample and of aPTT reagent followed by addition of an equal volume of pre-warmed 0.025 M calcium chloride solution to activate coagulation. Coagulation was determined spectrophotometrically.

### 2.15. Haemolysis Assay

To determine the effect of the formulations on blood, human erythrocytes were isolated from fresh blood as described previously [42]. Briefly, following prior consent from the donor, whole blood was collected into EDTA tubes as centrifuged to separate the red blood cell (RBC) pellet. The RBC pellet was washed thrice with PBS buffer (pH 7.4) and diluted to a ratio of 1:50 with PBS. The erythrocytes were incubated together with the formulations in V-bottom microtiter plates placed in an orbital shaker KS4000 IC (IKA Werke) for 1 h at 37 °C. Supernatants were collected from the plates following centrifugation and the absorbance was determined at 540 nm using a plate reader (FLUOstar Optima). PBS (pH 7.4) and 1% Triton X-100 were used as controls.

### 2.16. Statistical Analysis

All experiments were performed in triplicates and data are presented as mean ± standard deviation, unless otherwise stated. Two-tailed Student’s *t*-test was performed to identify statistical significance differences.

## 3. Results

### 3.1. Physicochemical Characterisation

Preliminary experiments were performed to determine an ideal stabiliser concentration, homogenisation speed and curcumin content. In emulsion–diffusion–evaporation technique, PVA acts as a stabiliser of the nanodroplets and is one of the widely used emulsifier for polymeric nanoparticle preparations. Both, particle size and size distribution are affected by the concentration of PVA in the aqueous phase during particle preparation [35]. After testing different PVA concentrations, we narrowed down to 2% PVA considering the physicochemical properties of the resultant nanoformulations. The Z-average data from DLS as well as LDA results are summarised in Table 1. CUR-NP and NP showed a narrow size distribution indicating a high reproducibility. No extensive change in particle size was noticeable after curcumin loading. This was also the case for lyophilised samples, thereby showing that lyophilisation has no deleterious effects on the physicochemical characteristics. Furthermore, LDA revealed a slightly negative ζ-potential at pH 7.4, regardless of curcumin loading for both formulations.

PLGA in an aqueous environment undergoes hydrolysis spanning over several weeks, depending on the ratio of lactic acid to glycolic acid. The PLGA 50:50 used in this study therefore exhibits the fastest degradation [43]. To increase the storage stability the nanoparticles were lyophilised, using PVA as cryoprotectant. Freeze-dried and resuspended nanoparticles showed no extensive change in particle size (CUR-NP +0.3 nm, NP −1.8 nm) or polydispersity index (data not shown).

For the determination of encapsulation efficiency, a direct method of dissolving the purified particles and measuring the actual amount of curcumin available in the nanoparticle formulation was used. Since curcumin is near to insoluble in water, un-encapsulated free curcumin should exist in crystalline form. In this case the crystals would sediment during ultracentrifugation and be present in the pellet. Hence discarding the supernatant would not lead to sufficient separation of nanoparticles from crystalline curcumin. Therefore (in contrast to NP), a first separation step of centrifugation with a relatively low force of 2000× *g* is necessary to sediment the larger crystals. The optimised formulation of CUR-NP showed a relatively high encapsulation efficiency (EE) with an actual loading capacity of 2% (Table 2). The good EE results can be explained by the fact that PLGA and curcumin are both soluble in ethyl acetate, which was employed for the nanoparticle preparation process. Moreover, PLGA’s ability to effectively encapsulate curcumin has been demonstrated in previous studies [44,45,46].

### 3.2. Morphological Characterisation

**Figure 1:**
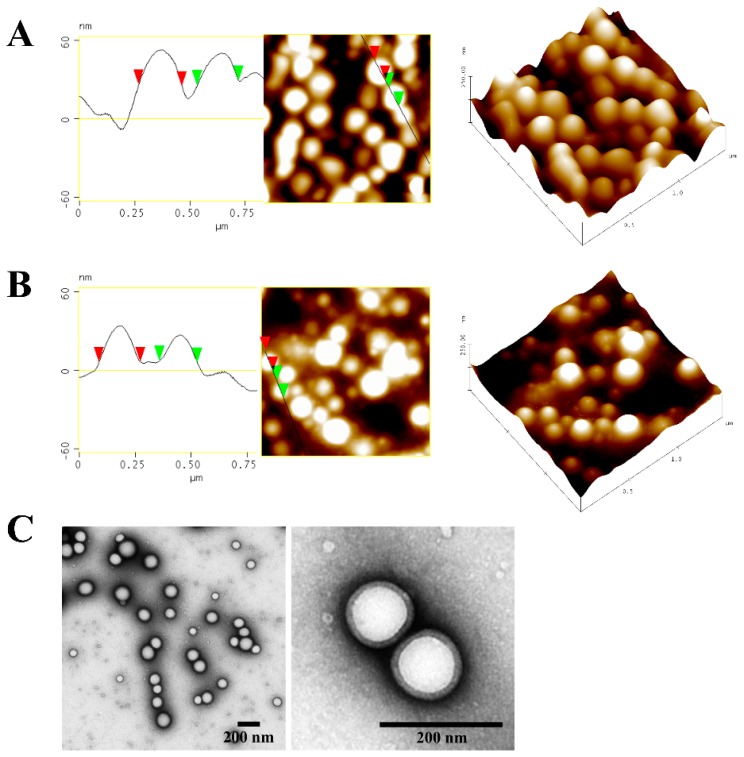
Morphology of representative curcumin-loaded poly(lactic-*co*-glycolic acid) (PLGA) nanoparticles (CUR-NP) shown by atomic force microscopy (AFM) (**A**,**B**) and transmission electron microscopy (TEM) (**C**). (**A**) Freshly prepared nanoparticles, (**B**,**C**) nanoparticles post lyophilisation. All AFM micrographs are presented in height mode. Samples were negatively stained with 2% uranyl acetate prior to TEM measurement. Scale bars in TEM micrographs represent 200 nm.

In all images (Figure 1), round-shaped CUR-NP with a monomodal size distribution are clearly visible. Due to dilution of pure nanoparticle samples, even single CUR-NPs with a smooth surface indicating that the curcumin is completely incorporated could be visualised. The AFM size analysis was in agreement with the DLS measurements. It should however be noted that the differences in size arising from the DLS and AFM measurements is because the hydrodynamic diameter is obtained in aqueous conditions whereas the latter is measured under atmospheric conditions [47]. Furthermore, a PVA corona was noticeable in TEM micrographs (Figure 1C). While free PVA was removed through centrifugation and washing of the redispersed nanoparticles prior to each experiment, it is a known fact, that small quantities of PVA still remain on the surface of PLGA nanoparticles, which cannot be removed even by extensive washing. This strong adsorption of PVA may be caused by hydrophobic bonding of PVA’s hydroxyl groups to the acetyl groups of PLGA [48].

### 3.3. In Vitro Drug Release

Different mechanisms were reported for the drug release from PLGA nanoparticles: (i) desorption of drug absorbed on the particles surface, (ii) diffusion through the polymer matrix, (iii) erosion of the polymer matrix, and (iv) a combination of erosion and diffusion processes [29,49].

**Figure 2:**
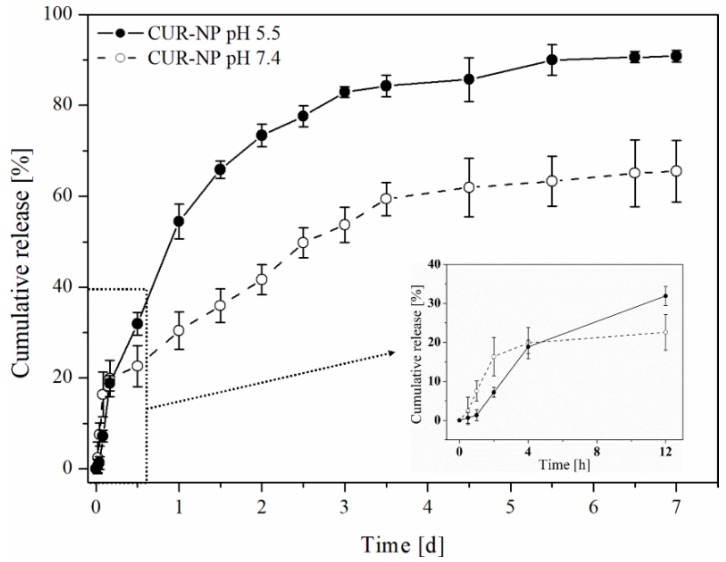
Cumulative in vitro drug release of curcumin from CUR-NP in phosphate buffer (pH 5.5 and 7.4) containing 1% polysorbate 80 to assure sink conditions. The inset represents the first 12 h of the release study in a different scale (hours instead of days).

The cumulative drug release of curcumin from CUR-NP at pH 5.5 and 7.4 is shown in Figure 2. Due to curcumin’s low water solubility (~0.01 μg/mL at pH 5 and ~0.4 μg/mL at pH 7.3) and low stability at neutral to basic pH, the addition of a solubility-enhancing component was necessary to assure sink conditions and to achieve UV/VIS detectable concentrations [50]. We chose polysorbate 80 for this purpose, since it is reported, that it is capable of increasing the solubility of hydrophobic drugs and protecting them against degradation through the formation of micelles [51].

CUR-NP showed a typical biphasic release pattern of PLGA nanoparticles with a burst release of around 20% loading within the initial 4 h, followed by controlled release of 90% drug loading over the following 7 days. Burst release is most likely attributed to the drug absorbed to the particle’s surface, while controlled release might be induced through a combination of erosion and diffusion processes [52,53]. Furthermore, drug release was much faster in acidic medium compared to pH 7.4 which could be caused by a faster degradation rate of PLGA under acidic conditions [33]. 

Tumour cells often exhibit an extracellular pH of around 6.5, which would lead to a faster release of curcumin in this acidic environment and therefore an increased cytotoxicity compared to healthy tissue [54,55]. Since the pH of endosomes is even lower (pH ~5.5), drug release of internalised nanoparticles would proceed even faster, leading to higher intracellular curcumin concentration and enhanced tumour damage [56]. Both mechanisms could be used for passive tumour-targeting strategy.

### 3.4. Serum Stability

Serum stability assay was performed to evaluate the susceptibility of the formulations. For this purpose, DLS and LDA analyses were performed after incubating the nanoparticles in serum. The experiments were carried at 37 °C in a shaking incubator to simulate physiological conditions. The results show shrinkage in the nanoparticle size after 24 h incubation time in serum (Table 3). This could be related to a release of curcumin bound to the particle surface, which leads to particle erosion [57]. Furthermore, the decrease in particle size could be caused by osmotic forces, as reported previously [58]. The increase in the PDI of the nanoparticles could be attributed to the decrease in homogeneity in the presence of serum. After an initial increase (as compared to DLS measurements from native particles), the ζ-potential decreased to more negative values. The initial increase could be due to the accumulation of plasma proteins on the surface of the NP and the subsequent decrease could be attributed to the drug release (curcumin) onto the surface of the NP [59]. 

### 3.5. Irradiation Experiments

**Figure 3:**
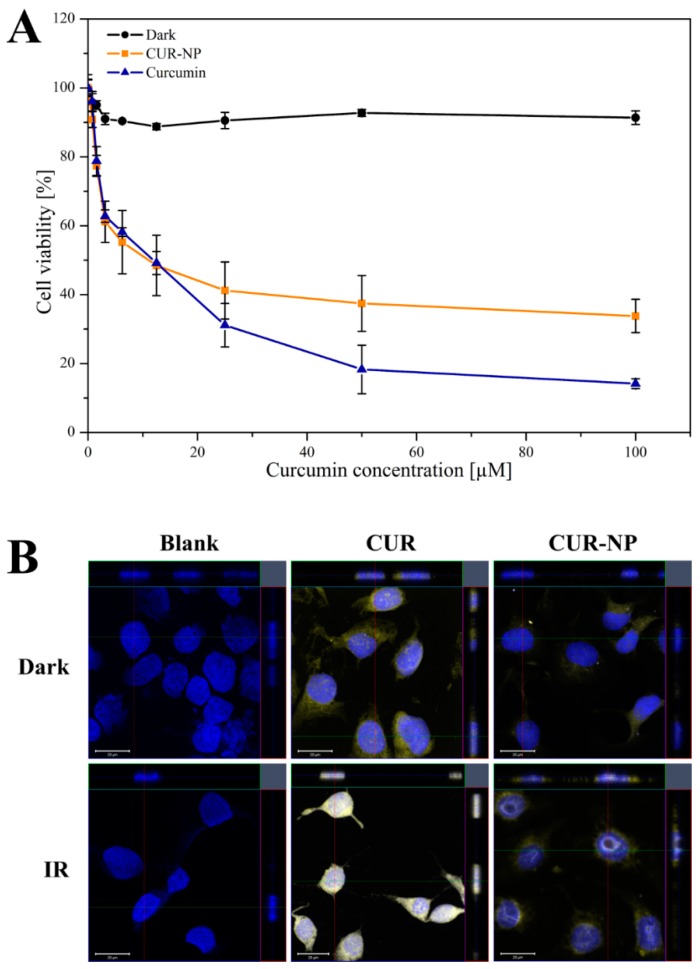
Phototoxic effect of curcumin-loaded poly(lactic-*co*-glycolic acid) PLGA nanoparticles (CUR-NP) and free curcumin dissolved in DMSO (CUR) on SK-OV-3 ovarian carcinoma cells: (**A**) for the MTT-assay, either the nanoformulation or free curcumin was incubated for 4 h at 37 °C and were irradiated at 457 nm with a radiation fluence of 8.6 J/cm^2^. Dark was used as negative control and represents cells without irradiation. All samples contain 0.1 mg/mL curcumin and were measured in triplicates (*n* = 9, independent formulations). Results are expressed as means ± SD. (**B**) CLSM micrographs of SK-OV-3 cells incubated with CUR-NP or free curcumin for 4 h at 37 °C and subsequent irradiation (457 nm, 8.6 J/cm^2^). The cell nucleus was counterstained with 0.1 µg/mL DAPI and was fixed with 4% formaldehyde solution. The curcumin concentration in CUR-NP was 50 µM. Nuclear damage is clearly witnessed in the irradiated samples whereas in the dark, the nucleus is intact. Dark was used as negative control and represents cells without irradiation. Scale bars denote 20 µm.

For the photo destructive effect of PDT, SK-OV-3 ovarian carcinoma cells were incubated with different concentrations of CUR-NP und free curcumin for 4 h and were irradiated with different radiation fluence. The efficacy of the irradiation was determined by MTT assay. As shown in Figure 3A, after treatment of cells, curcumin-loaded biodegradable nanoparticles and free curcumin dissolved in DMSO show a high photocytotoxic effect. There is a remarkable difference between the dark (non-irradiated) and irradiated plates. An incubation time of 4 h was found to be ideal among all the formulations, since burst release of around 20% curcumin loading from CUR-NP was found within 4 h of drug release studies (Figure 2). In the initial studies, a radiation fluence of 1.4, 4.3, 8.6 and 13.2 J/cm^2^ were tested using CUR-NP and for the subsequent studies, the fluence was narrowed down to 8.6 J/cm^2^ for the LED induced PDT (data not shown). Beyond a curcumin concentration of 50 µM, there was no further difference in the effect of PDT. It can also be seen that free curcumin dissolved in DMSO, induced a higher photocytotoxicity effect, which could be attributed to controlled release properties of PLGA nanoparticles, as this might be a rate limiting step for the drug being available for light activation. Nevertheless, since DMSO-dissolved curcumin is not suitable for therapeutic applications and due to its hydrophobic nature, we have used a biodegradable polymeric PLGA nanoformulation to increase the bioavailability of curcumin and to make it suitable for therapeutic applications [60]. The internalisation and subsequent localisation of the photosensitiser within the tumour determine the outcome of the therapy.

### 3.6. Subcellular Localisation of Curcumin

To visualise the effect of PDT on tumour cells, qualitative fluorescence microscopic analysis was performed after irradiation (457 nm; 8.6 J/cm^2^) of the cells with 50 µM CUR-NP and free curcumin dissolved in DMSO. The cells were fixed with 4% formaldehyde solution and the cell nucleus was counterstained with 300 nM DAPI. Substantial intracellular localisation of curcumin near the cell’s nucleus could be seen in both dark and irradiated samples, as shown in Figure 3B. Upon irradiation, photo-destruction induced by PDT could be clearly observed in the micrographs, which is evident from the nuclear perforation. This might be due to the chromatin condensation and DNA fragmentation caused by the curcumin induced PDT [8]. Comparing free curcumin dissolved in DMSO with curcumin-loaded nanoparticles reveals that CUR-NPs indeed damage the nucleus while only free curcumin alone is not as damaging although the micrographs show a cellular uptake of curcumin by both the samples. This confirms the hypothesis that curcumin particles are internalised by uptake mechanisms such as endocytosis and free curcumin penetrates the cells by diffusion and loses its effect (Section 3.6). It could also be seen that upon irradiation, the fluorescence of free curcumin increased and was more distributed than the CUR-NP. Furthermore, the irradiated blank/untreated cells show no photo-destruction.

### 3.7. Cellular Uptake

**Figure 4:**
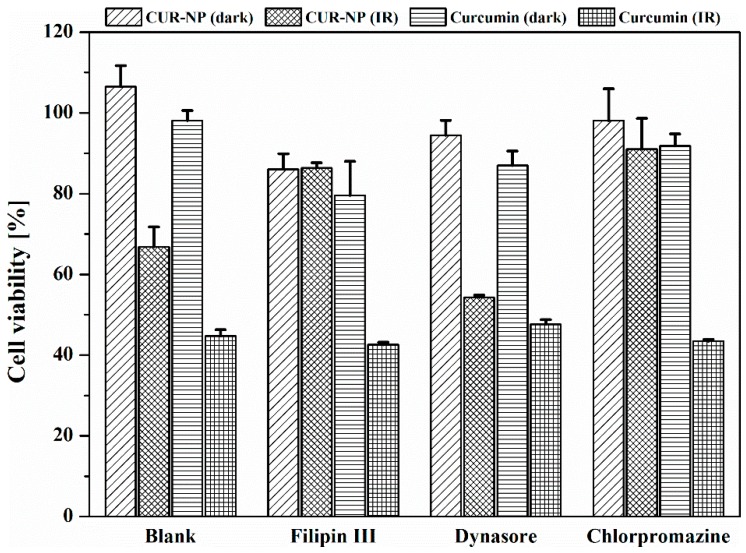
Cellular uptake of curcumin-loaded poly(lactic-*co*-glycolic acid) (PLGA) nanoparticles (CUR-NP) and free curcumin (dissolved in DMSO) in SK-OV-3 cells in presence of three specific inhibitors. After 30 min incubation time with different inhibitors (i.e., 8 µM Filipin III, 80 µM Dynasore and 14 µM chlorpromazine), cells were incubated with 50 µM of either CUR-NP or free curcumin dissolved in DMSO for 4 h at 37 °C and were irradiated at 457 nm with a radiation fluence of 8.6 J/cm^2^ (IR). The MTT assay was performed at the end of the experiment to determine the effect of the pathway inhibits on internalisation. Viability of untreated cells was considered as 100%. Dark was used as negative control and represents unirradiated samples. Blank represents cells without any inhibitor. All samples were measured in triplicates (*n* = 9, independent formulations) and results are expressed as means ± SD.

Different cells use different endocytic pathways for the internalisation of nanoparticles. To study cellular uptake, several different inhibitors of endocytic pathways were utilised. For this purpose, the dynamin-, clathrin- and clathrin-independent endocytosis pathways were considered. Dynasore, a GTPase inhibitor rapidly and reversibly inhibits dynamin activity, which is essential for membrane fission during clathrin-mediated endocytosis (CME). Chlorpromazine also causes a block in CME by inducing the assembly of adaptor proteins and clathrin, leading to the formation of endosomes which then fuse with lysosomes [61]. Chlorpromazine also interferes with the intracellular clathrin processing [62]. Filipin III derived from *Streptomyces filipensis* is a polyene macrolide antibiotic, which inhibits the raft/caveolae endocytosis pathway. It interacts with cholesterol whose presence and state influence endocytic functions [63]. Figure 4 shows a substantial inhibition of CUR-NP by Filipin III and chlorpromazine. The uptake mechanism is dependent on the cell line and hence the data should be regarded cautiously. In our case, chlorpromazine exhibited an 80–90% inhibition of clathrin-dependent endocytosis of CUR-NP and Filipin III a marked 100% inhibition of caveolae-mediated and lipid-raft mediated endocytosis but both could not inhibit free curcumin. Free curcumin dissolved in DMSO does not seem to be internalised by any of these endocytic pathways suggesting a different internalisation mechanism such as diffusion [64]. The inhibition by dynamin dependant endocytosis by Dynasore showed no effect on the CUR-NP.

### 3.8. Reactive Oxygen Species

**Figure 5:**
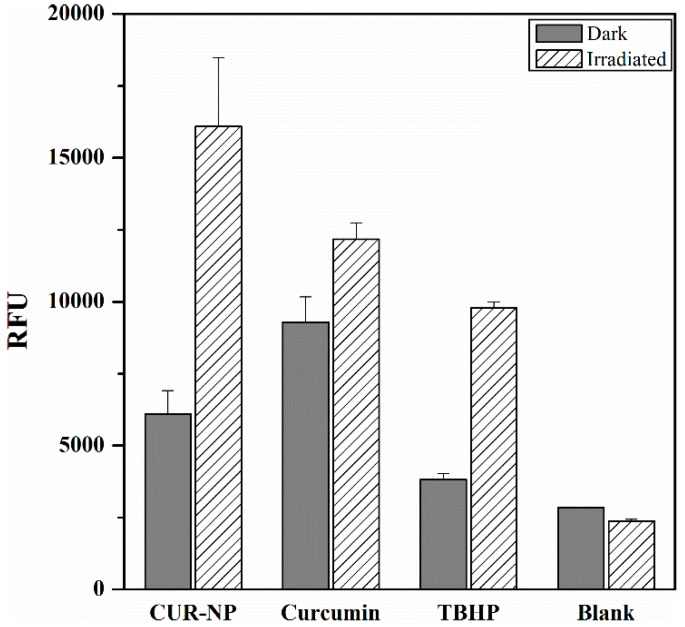
Production of cellular reactive oxygen species (ROS) in response to curcumin-loaded poly(lactic-*co*-glycolic acid) (PLGA) nanoparticles (CUR-NP) or free curcumin (0.1 mg/mL). ROS was measured in SK-OV-3 cells after exposure (for 45 min) to 2’,7’-dichlorofluorescin diacetate (25 µM) and incubation with CUR-NP or free curcumin for 4 h at 37 °C with subsequent irradiation (457 nm, 8.6 J/cm^2^). Tert-butyl hydroperoxide (TBHP) was used as positive control and non-irradiated cells served as negative control (Dark), whereas Blank represents untreated cells. Values are represented in relative fluorescence units (RFU). All samples were measured in triplicates (*n* = 9, independent formulations) and results are expressed as means ± SD.

ROS generation is the backbone of PDT and is a determining factor in the efficacy of the therapy. As shown in Figure 5, curcumin induced the production of cytotoxic reactive oxygen species after cells were irradiated (50 µM curcumin; 8.6 J/cm^2^ radiation fluence). ROS was determined by the measurement of the fluorescence from the lysed cells. Upon incubation with cells, a deacetylated form of DCFDA is oxidised by the resulting ROS to form fluorescent DCF [65]. As expected, curcumin-loaded biodegradable PLGA polymeric nanoparticles exhibited the highest ROS generation followed by free curcumin dissolved in DMSO thereby corresponding to the results of the PDT. The fact that CUR-NP caused the maximum damage to the tumour cells can be explained by the ROS generation, which in this case was intracellular and could be only detected from the cells, which have internalised. Since free curcumin was not internalised by the endocytic pathways, mentioned in Figure 4, the uptake from CUR-NP seems to be higher. As expected, blank (negative control) did not show any ROS generation.

### 3.9. Haemocompatibility

**Figure 6:**
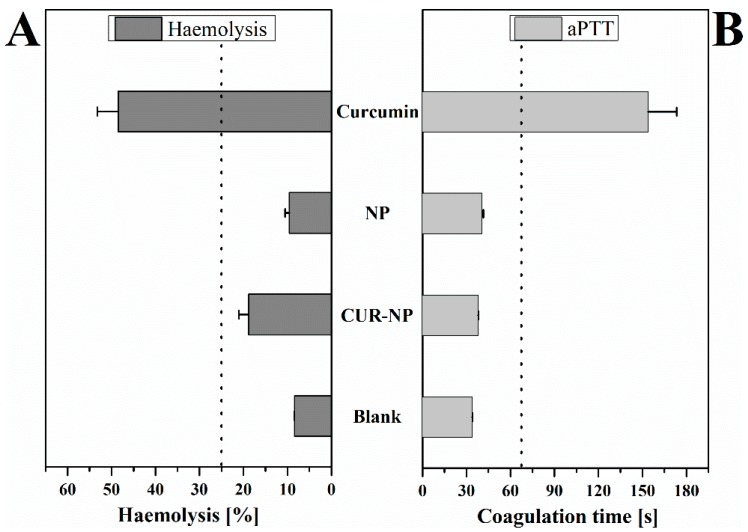
Blood compatibility of curcumin-loaded poly(lactic-*co*-glycolic acid) (PLGA) nanoparticles (CUR-NP), unloaded PLGA nanoparticles (NP) or free curcumin: (**A**) haemolysis assay and (**B**) aPTT test were performed. Where applicable, curcumin was used at a concentration of 0.1 mg/mL. Blank represents erythrocytes in the haemolysis assay and blood plasma in the aPTT test, respectively. Triton X-100 was used as positive control in (**A**) and is considered as 100% haemolysis. All samples were measured in triplicates (*n* = 9, independent formulations) and results are expressed as means ± SD. Dotted lines indicate the tolerance/threshold limits.

To study the effect of the nanoformulation on human erythrocytes, haemolysis assay, which determines the amount of haemoglobin released from the erythrocytes upon exposure to NPs (Figure 6A) was performed. To determine the change in coagulation time upon addition of NPs, aPTT was determined (Figure 6B). Upon its release from erythrocytes, haemoglobin reacts with the atmospheric oxygen to form oxyhaemoglobin [66]. The CUR-NP used in the study showed a minimal haemolytic potential. An increase in the aPTT time was also found to be minimal in the case of CUR-NP. The aPTT analysis revealed that the encapsulated curcumin increased the coagulation time by only 4.01 s, whereas upon addition of free curcumin alone, the coagulation time increased to 120.13 s suggesting an interaction of curcumin with the intrinsic proteins or coagulation factors. The normal coagulation time for the plasma was tested to be 32.2 ± 0.1 s and was found to be in the normal range [67]. Coagulation time between 30 and 40 s is considered within acceptable range and coagulation time above 70 s denotes spontaneous and continuous bleeding leaving the patients with the risk of haemorrhage [67,68,69].

## 4. Conclusions

In this study, curcumin-loaded biodegradable PLGA nanoparticles (CUR-NP) were formulated for photodynamic therapy using a custom manufactured prototype low power LED device. CUR-NP exhibited better physicochemical properties compared to free curcumin, such as improved serum stability. Furthermore, haemocompatibility of curcumin was improved through polymeric encapsulation, minimising the risk of haemorrhages in patients. Successful cellular uptake of CUR-NP was demonstrated with the aid of confocal laser scanning microscopy, which showed photo-destruction and nuclear perforation. Nanoformulation facilitated the use of higher amounts of curcumin, which would otherwise be impossible due to its poor solubility and stability in aqueous media. Curcumin, which elsewise is safe for normal cells, showed cytotoxic effects on tumour cells upon irradiation at a low intensity therefore selectively inhibiting tumour growth. Since production of ROS occurs only upon irradiation of the intracellular photosensitiser, tumour tissues can be targeted with precision. Using LED as an irradiation source offers a number of advantages such as portability, durability and economical compared to lasers. Moreover, LED’s have the ability to irradiate a relatively larger area, which could be beneficial for the treatment of large tumours or even skin cancer. The use of visible light of low-energy makes this therapy safer for both the patient and the operator. Finally, the good biocompatibility of this well-documented and highly reproducible formulation process of polymeric nanoparticles are significant advantages of this anti-tumour therapy. Transforming this novel strategy into a safe and effective therapy by combining PDT with other therapies would be our prime research interest in the future.

## Figures and Tables

**Table 1:** Hydrodynamic diameter, polydispersity index (PDI) and ζ-potential of curcumin-loaded poly(lactic-*co*-glycolic acid) (PLGA) nanoparticles (CUR-NP) and unloaded PLGA nanoparticles (NP). Lyophilised samples represent samples resuspended after lyophilisation. Hydrodynamic diameters are expressed as a measure of particle size distribution by intensity. All samples were measured in triplicates (*n* = 9, independent formulations) and results are expressed as means ± SD.

Sample	Diameter [nm]	PDI	ζ-potential [mV]
NP	194.7 ± 8.7	0.09 ± 0.05	−5.33 ± 0.88
CUR-NP	203.6 ± 7.8	0.08 ± 0.04	−5.24 ± 0.86
Lyophilised NP	195.0 ± 6.9	0.09 ± 0.05	−5.09 ± 0.73
Lyophilised CUR-NP	201.8 ± 6.0	0.09 ± 0.03	−5.43 ± 0.67

**Table 2:** Theoretical load, percentage of yield, encapsulation efficiency and loading capacity of curcumin-loaded poly(lactic-*co*-glycolic acid) (PLGA) nanoparticles (CUR-NP) and unloaded PLGA nanoparticles (NP). All samples were measured in triplicates (*n* = 9, independent formulations) and results are expressed as means ± SD.

Sample	Theoretical Load [%] ^a^	%Yield	%EE	%LC
NP	-	80.5 ± 2.3	-	-
CUR-NP	2.5	87.5 ± 0.7	80.4 ± 10.6	2.0 ± 0.3

^a^ % *w*/*w* loading of curcumin to polymer; EE, encapsulation efficiency; LC, loading capacity.

**Table 3:** Physicochemical changes of poly(lactic-*co*-glycolic acid) (PLGA) nanoparticles containing 0.1 mg/mL curcumin. Particles were incubated for 24 h in serum at a volume ratio of 5:1. The hydrodynamic diameter is expressed as a measure of particle size distribution by intensity. All samples were measured in triplicates (*n* = 9, independent formulations) and data are expressed as means ± SD.

Time [h]	Diameter [nm]	PDI	ζ-potential [mV]
0	183.50 ± 2.11	0.19 ± 0.01	2.19 ± 0.49
1	180.80 ± 3.70	0.19 ± 0.01	−8.30 ± 0.21
4	180.45 ± 3.32	0.22 ± 0.01	−12.65 ± 0.35
24	173.57 ± 2.21	0.22 ± 0.01	−13.30 ± 0.10
